# WWP1 mediates the ubiquitination and degradation of HIPK3 in bladder cancer cells

**DOI:** 10.1016/j.jbc.2025.108528

**Published:** 2025-04-23

**Authors:** Haichao Chen, Xinyu Na, Pengcheng Hu, Qi Ma, Rui Yu

**Affiliations:** 1 Department of Urology, The First Affiliated Hospital of Ningbo University, Ningbo, Zhejiang Province, China; 2 Department of Urology, Zhenhai People’s Hospital, Ningbo, Zhejiang Province, China; 3 Department of Biochemistry and Molecular Biology, School of Basic Medical Sciences, Health Science Center, Ningbo University, Ningbo, Zhejiang Province, China

**Keywords:** HIPK3, WWP1, ubiquitination, JNK

## Abstract

Protein homeostasis is primarily regulated by post-translational modifications (PTMs). HIPK3 has been recognized as a tumor suppressor across various cancers. However, the impact of PTMs on HIPK3 remains insufficiently explored. This study identified WWP1 as an E3 ubiquitin ligase targeting HIPK3, demonstrating that WWP1 downregulates HIPK3 protein levels by facilitating its ubiquitination. Mechanistically, WWP1 directly interacts with HIPK3, promoting K48-linked polyubiquitination at the K1187 site. The WWP1/HIPK3 axis modulates cancer cell chemosensitivity through the regulation of the JNK signaling pathway. Additionally, Myc was found to act as a transcription factor, enhancing WWP1 expression. These findings offer novel insights into the regulation of HIPK3 at the PTM level.

Bladder cancer (BCa), an aggressive tumor of the genitourinary system, ranks as the 10th most commonly diagnosed cancer worldwide (1). Several factors, including smoking, alcohol consumption, and obesity, contribute to the progression of BCa (2). Despite advancements in surgical and adjuvant treatments, the prognosis for patients with BCa remains poor (3). Therefore, understanding the underlying mechanisms of BCa tumorigenesis and developing potential treatment strategies is essential.

The homeodomain-interacting protein kinase (HIPK) family comprises four evolutionarily conserved serine/threonine kinases: HIPK1-4 (4). HIPK1-3 are structurally related, containing an N-terminal kinase region and multiple other domains that facilitate protein interactions (5). For instance, HIPK3 interacts with FADD, mediating its phosphorylation and inhibiting Fas-induced JNK signaling activation (6). Increasing evidence suggests that HIPK3 acts as a tumor suppressor in various cancers. For example, reduced HIPK3 expression correlates with poor survival in patients with clear cell renal cell carcinoma (7). Additionally, low HIPK3 levels contribute to cisplatin resistance in gastric cancer, with overexpression of HIPK3 overcoming this resistance (8). Furthermore, HIPK3 downregulation is significantly associated with poor prognosis in other cancers (9). However, the mechanisms regulating HIPK3 protein levels remain unclear.

In eukaryotic cells, protein homeostasis is regulated by a complex protein degradation network. Two canonical systems involved in protein degradation are the autophagy-lysosome system and the ubiquitin-proteasome system (10). Ubiquitination, a common post-translational modification (PTM), tags target proteins with ubiquitin, leading to their degradation by the 26S proteasome complex (11). Ubiquitination is tightly regulated by three core enzymes: the ubiquitin-activating enzyme (E1), ubiquitin-conjugating enzyme (E2), and ubiquitin-protein ligase (E3) (11). E3 ligases recognize specific substrates and mediate the transfer of ubiquitin to lysine residues on target proteins. To date, over 600 E3 ligases have been identified in humans, categorized into three groups based on their ubiquitination domains: RBR (RING-Between-RING), RING (Really Interesting New Gene), and HECT (Homologous to E6AP C-Terminus) domain E3 ligases (12). WWP1 is a HECT E3 ligase with an N-terminal C2 domain, four tandem WW domains, and a C-terminal catalytic HECT domain (13). WWP1 functions as an oncogene in various cancers, with high levels often correlating with poor prognosis (14). For example, WWP1 upregulation is associated with poor prognosis and promotes colorectal cancer progression (15). WWP1 also facilitates breast cancer cell tumorigenesis by mediating the degradation of ErbB4, a tumor suppressor in breast cancer (16). However, the role and targets of WWP1 in BCa remain largely unexplored.

This study identified WWP1 as an interaction partner of HIPK3. WWP1 promotes the ubiquitination and subsequent degradation of HIPK3. Our findings reveal a previously unrecognized mechanism underlying the downregulation of HIPK3 in BCa cells.

## Results

### WWP1 interacts with HIPK3

**Figure 1:**
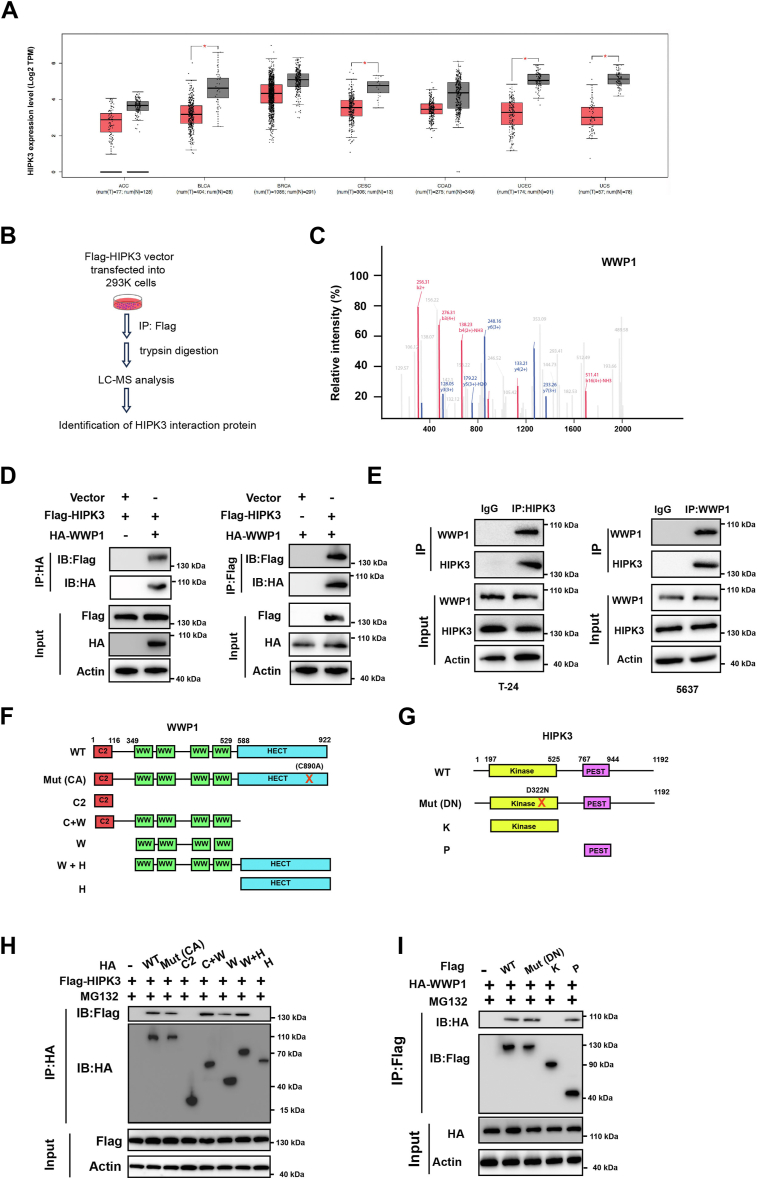
**Identification of WWP1 as the HIPK3-interacting protein**. *A*, the expression of HIPK3 across various cancers was analyzed using the GEPIA database (http://gepia.cancer-pku.cn/). Cancers studied include BLCA (bladder carcinoma), CESC (cervical carcinoma), UCS (uterine carcinosarcoma), UCEC (endometrial carcinoma), THYM (thymoma), LUAD (lung adenocarcinoma), and TGCT (testicular tumor). *B*, experimental procedure for identifying HIPK3 binding proteins. 293K cells were transfected with a vector expressing Flag-tagged HIPK3 or an empty vector. Cellular lysates underwent immunoprecipitation with anti-Flag antibody, followed by LC-MS/MS analysis. *C*, mass spectrometric identification of WWP1. *D*, 293K cells were transfected as indicated, followed by IP with HA- or Flag-conjugated beads and subsequent SDS-PAGE analysis with the indicated antibodies. *E*, Western blotting analysis of reciprocal Co-IP analysis in T24 and 5637 cells, confirming endogenous interaction between HIPK3 and WWP1. *F*, schematic representation of WWP1 constructs. *G*, schematic representation of HIPK3 constructs. *H*, 293K cells were transfected with Flag-HIPK3 alone or with HA-tagged full-length or mutant WWP1 constructs or domains, as indicated. Cellular lysates were immunoprecipitated with anti-HA antibody and subjected to SDS-PAGE analysis. *I*, 293K cells were transfected with HA-WWP1 alone or with Flag-tagged full-length or mutant HIPK3 constructs or domains, as indicated. Cellular lysates were immunoprecipitated with anti-Flag antibody and subjected to SDS-PAGE analysis.

Analysis of the TCGA dataset revealed downregulation of HIPK3 in BCa and various other malignancies (Fig. 1*A*). To investigate the underlying molecular mechanism, potential HIPK3-binding proteins were examined. 293T cells were transfected with either an empty vector or a FLAG-tagged HIPK3-expressing vector, followed by immunoprecipitation with an anti-FLAG antibody and LC-MS/MS analysis of the immune complex (Fig. 1*B*). WWP1 emerged as the top candidate, given its function as an E3 ligase (Fig. 1*C* and Supplementary Dataset 1). To confirm the interaction between WWP1 and HIPK3, co-IP and Western blotting analyses were conducted. HA-tagged WWP1 was co-transfected with FLAG-tagged HIPK3 into 293T cells, demonstrating a direct interaction between WWP1 and HIPK3 (Fig. 1*D*). Furthermore, endogenous WWP1 was found to bind HIPK3 in BCa cells under physiological conditions (Fig. 1*E*). To dissect the interaction further, FLAG-tagged HIPK3 and HA-tagged WWP1 domain constructs were expressed in 293T cells. The domains of WWP1 and HIPK3 are depicted in Figure 1, *F* and *G*. A point mutation (C890 A) was introduced in the HECT domain of WWP1 to disrupt its catalytic activity (Fig. 1*F*), while a D322 N mutation in the kinase domain of HIPK3 was employed to impair its kinase function (Fig. 1*G*). Co-IP assays revealed that the interaction between WWP1 and HIPK3 requires the WW (1–4) domains of WWP1 (Fig. 1*H*). Additionally, the PEST (proline-glutamic acid-serine-threonine) domain of HIPK3 was identified as essential for binding with WWP1 (Fig. 1*I*). Notably, the interaction between HIPK3 and WWP1 was unaffected by the inactive point mutations in the HECT or kinase domains (Fig. 1, *H*-*I*). Consequently, neither the E3 ligase activity of WWP1 nor the kinase activity of HIPK3 is necessary for their interaction. Instead, the physical binding is governed by the WW domain of WWP1 and the PEST domain of HIPK3.

### WWP1 negatively regulates the stability of HIPK3

**Figure 2:**
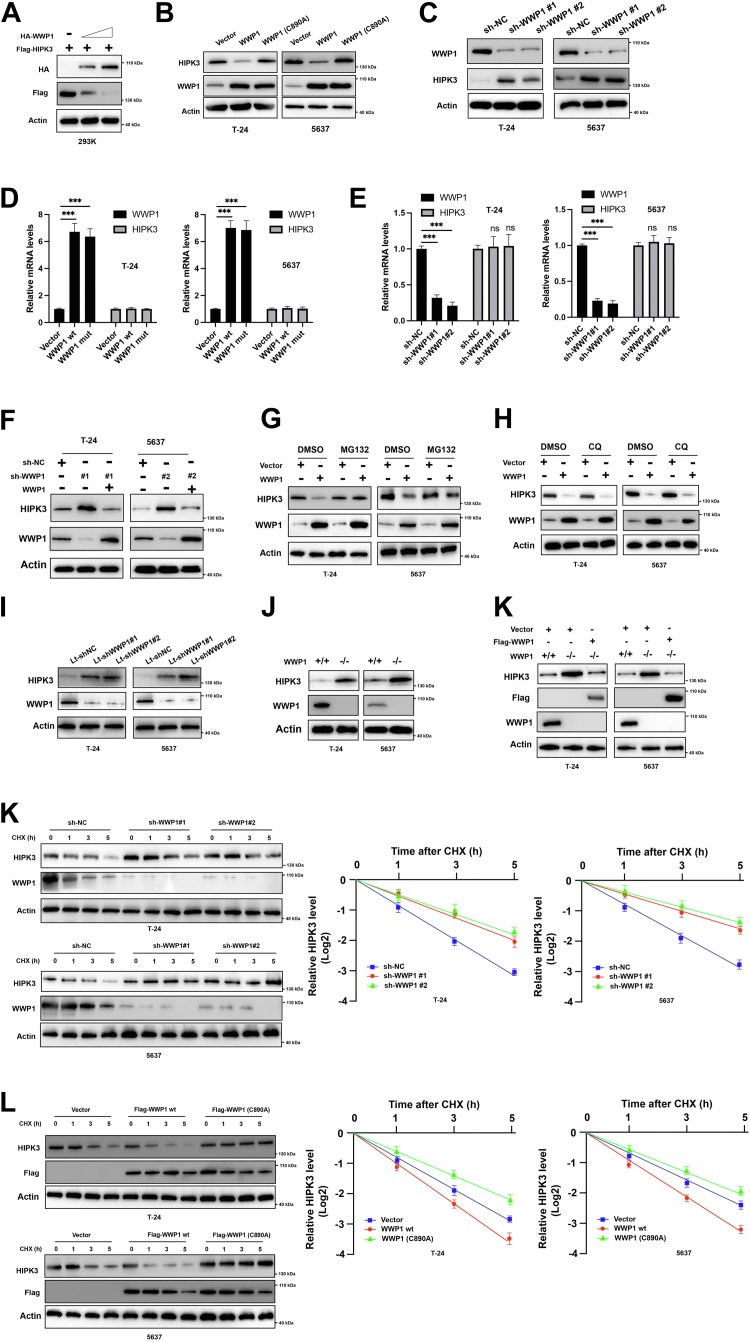
**WWP1 negatively regulates the protein levels of HIPK3**. *A*, 293K cells were transfected with Flag-HIPK3 alone or in combination with increasing amounts of HA-WWP1. Total cellular lysates were analyzed by Western blotting using the indicated antibodies. *B*, T-24 and 5637 cells were transfected with either an empty vector or vectors expressing wild-type or mutant WWP1. Cellular lysates were subjected to Western blotting with the indicated antibodies. *C*, T-24 and 5637 cells were transfected with negative control shRNA or shRNAs targeting WWP1. Total cellular lysates were analyzed by Western blotting using the indicated antibodies. *D* and *E*, T-24 and 5637 cells were transfected as indicated, and the relative mRNA levels of WWP1 and HIPK3 were quantified by RT-PCR. *F*, T-24 and 5637 cells were transfected with shWWP1 alone or in combination with a vector expressing WWP1. Total cellular lysates were analyzed by Western blotting with the indicated antibodies. *G*, T-24 and 5637 cells were transfected with an empty vector or WWP1 overexpression vector in the presence or absence of MG132. Total cellular lysates were analyzed by Western blotting. *H*, T-24 and 5637 cells were transfected with an empty vector or WWP1 overexpression vector in the presence or absence of CQ. Total cellular lysates were analyzed by Western blotting. *I*, T-24 and 5637 cells were transduced with lentiviruses expressing sh-NC or sh-WWP1. Total cellular lysates were analyzed by Western blotting. *J*, WWP1 was depleted by CRISPR/Cas9 in T-24 and 5637 cells. Total cellular lysates were analyzed by Western blotting. *K*, WWP1 (+/+) BCa cells were transfected with an empty vector, and WWP1 (−/−) BCa cells were transfected with either an empty vector or Flag-WWP1 vector. Total cellular lysates were analyzed by Western blotting. *L*, T-24 and 5637 cells were transfected with sh-NC or sh-WWP1. Cells were treated with cycloheximide (CHX) for the indicated time periods. Total cellular lysates were analyzed by Western blotting (*Left*). Band densities were quantified using ImageJ, and decay curves are shown on the *right*. *M*, T-24 and 5637 cells were transfected with an empty vector or vectors expressing wild-type or mutant WWP1. Cells were treated with CHX for the indicated time periods. Total cellular lysates were analyzed by Western blotting (*Left*).

Our results indicate that WWP1 may function as an E3 ligase, negatively regulating HIPK3 protein levels. To confirm this, WWP1 was overexpressed in 293K cells, leading to a dose-dependent downregulation of HIPK3 (Fig. 2*A*). To assess whether this inhibition depends on WWP1's enzymatic activity, both wild-type (wt) WWP1 and the inactive C890 A mutant were transfected into 5637 and 293K cells. As shown in Figure 2*B*, only wt WWP1, not the C890 A mutant, reduced HIPK3 protein levels in BCa cells. Additionally, WWP1 knockdown resulted in HIPK3 accumulation in these cells (Fig. 2*C*). Notably, WWP1 overexpression or downregulation did not affect HIPK3 mRNA levels (Fig. 2, *D* and *E*). Furthermore, overexpression of WWP1 reversed the effect of WWP1 silencing on HIPK3 protein levels (Fig. 2*F*). Treatment with the proteasome inhibitor MG132 counteracted the WWP1-mediated reduction of HIPK3 (Fig. 2*G*), while the autophagy inhibitor CQ had no such effect (Fig. 2*H*). Lentivirus-mediated knockdown of WWP1 also led to HIPK3 accumulation in BCa cells (Fig. 2*I*). CRISPR/Cas9 was used to knock out WWP1 in BCa cells (Fig. 2*J*), revealing that HIPK3 levels were elevated in WWP1-deleted BCa cells compared to wild-type cells (Fig. 2*J*). Reintroduction of WWP1 in these cells restored the reduction of HIPK3 (Fig. 2*K*). To further investigate, cycloheximide (CHX) was used to inhibit new protein synthesis, showing that silencing WWP1 significantly prolonged the half-life of HIPK3 proteins in BCa cells (Fig. 2*K*). Overexpression of wt WWP1, but not the inactive C890 A mutant, significantly shortened the half-life of HIPK3 proteins (Fig. 2*J*). Collectively, these results suggest that WWP1 negatively regulates the protein stability of HIPK3 in BCa cells.

### WWP1 mediates the ubiquitination of HIPK3 protein

**Figure 3:**
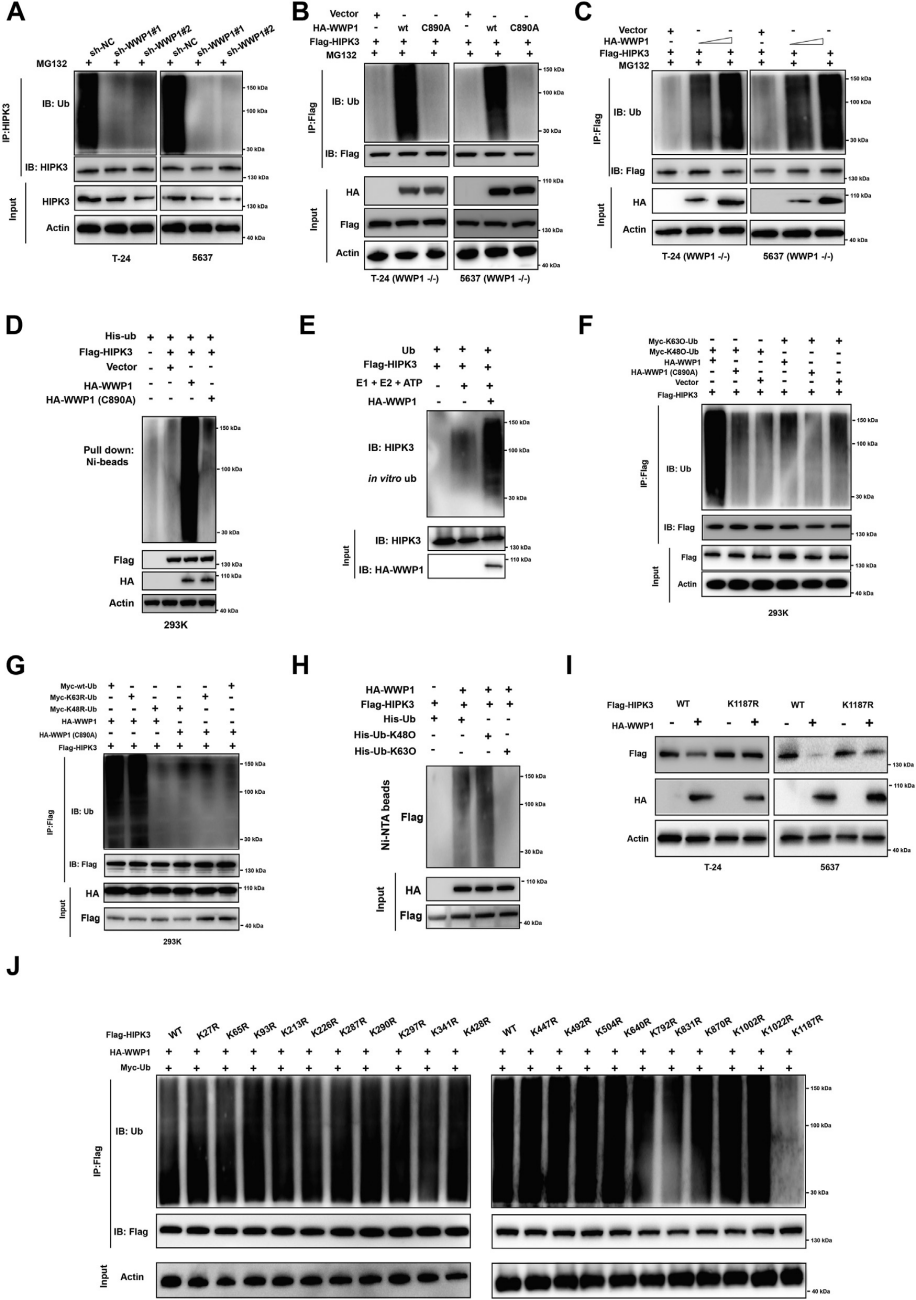
**WWP1 enhances HIPK3 ubiquitination**. *A*, T-24 and 5637 cells were transfected with sh-NC or shRNA targeting WWP1. The ubiquitination status of endogenous HIPK3 was examined, with input analyzed by Western blotting using the indicated antibodies. *B*, T-24 WWP1 KO and 5637 WWP1 KO cells were transfected with Flag-HIPK3 alone or with HA-tagged wild-type or mutant WWP1. Cellular lysates were pulled down using anti-Flag beads and immunoblotted with anti-Ub and anti-Flag antibodies. Input was analyzed by Western blotting with the indicated antibodies. *C*, T-24 WWP1 KO and 5637 WWP1 KO cells were transfected with Flag-HIPK3 alone or with increasing amounts of HA-tagged WWP1. Cellular lysates were pulled down with anti-Flag beads and immunoblotted with anti-Ub and anti-Flag antibodies. Input was analyzed by Western blotting with the indicated antibodies. *D*, 293K cells were transfected as indicated, followed by lysis under denaturing conditions (6M guanidine solution), Ni-beads pull-down, washing, and boiling. The polyubiquitination of HIPK3 was detected by Western blotting. *E*, *In vitro* ubiquitination assays were performed using commercial E1, E2, and Ub proteins, bacterially purified Flag-HIPK3, and WWP1 purified from WWP1-overexpressing HEK293 T cells. Reaction products were subjected to immunoblotting. *F*, 293K cells were transfected as indicated, followed by pull-down with anti-Flag beads and immunoblotting with anti-Ub and anti-Flag antibodies. Input was analyzed by Western blotting with the indicated antibodies. *G*, 293 K cells were transfected as indicated, followed by pull-down with anti-Flag beads and immunoblotting with anti-Ub and anti-Flag antibodies. Input was analyzed by Western blotting with the indicated antibodies. *H*, The *in vitro* ubiquitination assay was conducted, followed by Western blotting analysis. *I*, T-24 and 5637 cells were transfected with HA-tagged WWP1 in combination with Flag-tagged wild-type HIPK3 or K1187 R HIPK3. Total cellular lysates were analyzed by Western blotting with the indicated antibodies. *J*, 293K cells were transfected with wild-type HIPK3 or mutants, together with WWP1 and ubiquitin for 24 h. The ubiquitination status of HIPK3 was examined by immunoprecipitation followed by Western blotting analysis.

To determine whether WWP1 catalyzes the ubiquitination of HIPK3, WWP1 was silenced in BCa cells, followed by treatment with MG132. HIPK3 was then immunoprecipitated, and its ubiquitination status was assessed by Western blotting. Downregulation of WWP1 significantly reduced HIPK3 polyubiquitination in BCa cells (Fig. 3*A*). Next, wild-type WWP1 or the inactive mutant WWP1 were overexpressed in WWP1 −/− BCa cells, revealing that wild-type WWP1 significantly enhanced HIPK3 polyubiquitination, unlike the inactive mutant (Fig. 3*B*). Additionally, ectopic expression of WWP1 increased HIPK3 ubiquitination in a dose-dependent manner (Fig. 3*C*). To further confirm that WWP1 regulates HIPK3 ubiquitination, Flag-HIPK3 and His-Ub were co-transfected into 293K cells with either wild-type or inactive WWP1 (C890 A). Ni-NTA denaturing pull-down assays demonstrated that wild-type WWP1 significantly promoted HIPK3 ubiquitination, while the inactive mutant did not (Fig. 3*D*). In agreement, *in vitro* ubiquitination assays indicated that WWP1 facilitates HIPK3 ubiquitination (Fig. 3*E*). To investigate the type of ubiquitin linkage, six of the seven lysine residues of ubiquitin were mutated to arginine (*e*.*g*., K48O or K63O). Flag-HIPK3 and HA-WWP1 (wild-type or inactive) were co-transfected into 293K cells with Myc-K63O-Ub or Myc-K48O-Ub plasmids. Immunoprecipitation and subsequent Western blotting analysis revealed that wild-type WWP1, but not the inactive mutant, promoted K48-linked ubiquitination of HIPK3 (Fig. 3*F*). To further verify this, K48 R/K63 R Ub plasmids were used, where only the Lys48 or Lys63 residue was mutated to arginine. The K48 R Ub failed to link with Flag-HIPK3 under WWP1 overexpression (Fig. 3*G*). Moreover, Ni-NTA pull-down followed by Western blotting confirmed that WWP1 predominantly facilitates K48-linked ubiquitination of HIPK3 (Fig. 3*H*). The HIPK3 K1187 R mutant exhibited resistance to WWP1-mediated degradation (Fig. 3*I*). The lysine sites on HIPK3 that could be modified by WWP1-regulated ubiquitination were investigated. Considering that HIPK3 contains 20 potential lysine sites for ubiquitin attachment, 20 mutants were generated, each with a lysine-to-arginine substitution. Ubiquitination analysis revealed that WWP1 predominantly facilitated HIPK3 ubiquitination at the K1187 site (Fig. 3*J*). These results confirm that WWP1 acts as an E3 ligase, facilitating the ubiquitination of HIPK3 at the K1187 site.

### WWP1 and HIPK3 coordinately regulate the proliferation of BCa cells

**Figure 4:**
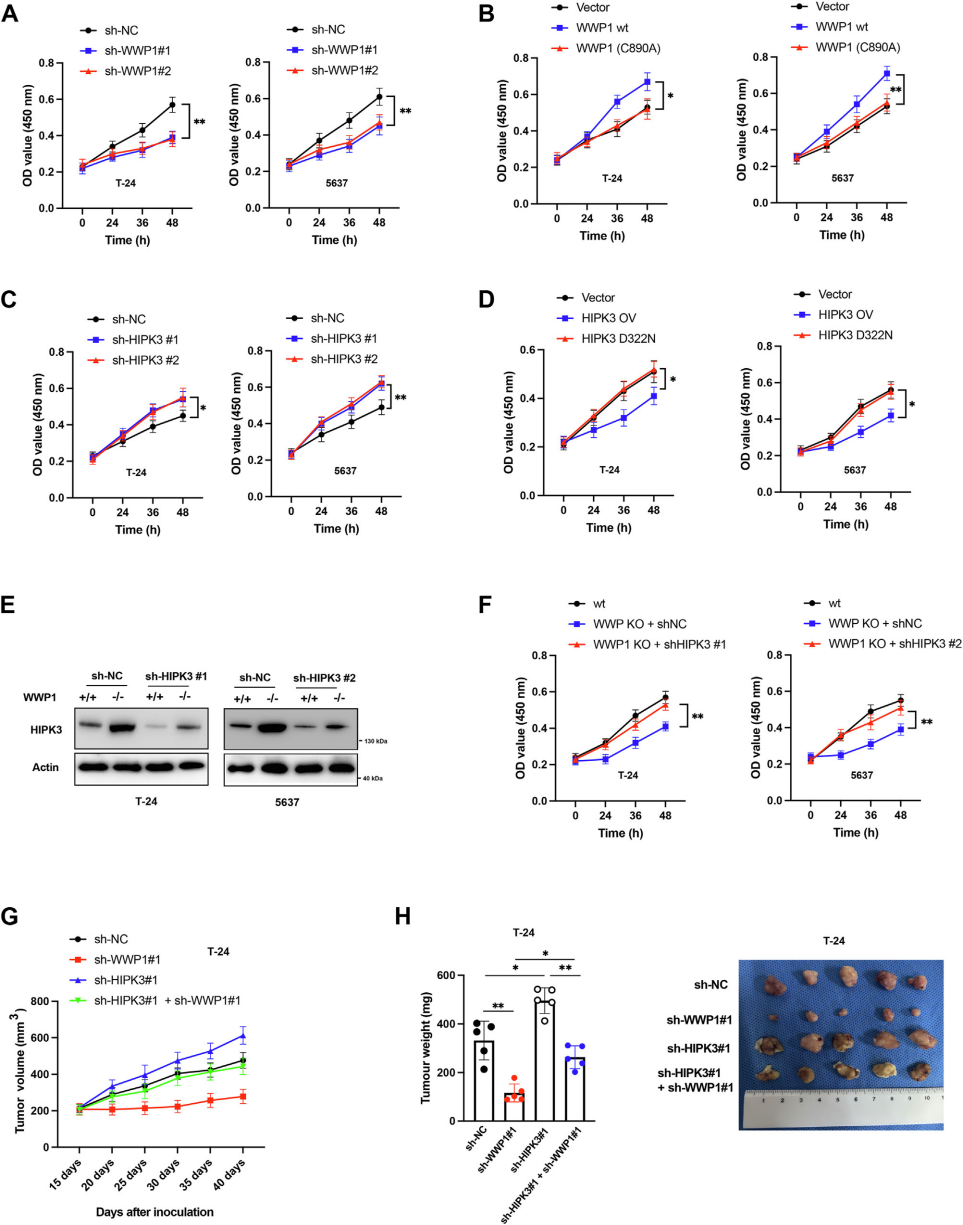
**WWP1/HIPK3 axis regulates BCa cell viability**. *A*, T-24 and 5637 cells were transfected with sh-NC or shRNAs targeting WWP1, and cell viability was measured at different time points. *B*, T-24 and 5637 cells were transfected with an empty vector, WWP1 overexpression vector, or WWP1 mutant overexpression vector. Cell viability was assessed at various time points. *C*, T-24 and 5637 cells were transfected with sh-NC or shRNAs targeting HIPK3, and cell viability was measured at different time points. *D*, T-24 and 5637 cells were transfected with an empty vector, HIPK3 overexpression vector, or HIPK3 mutant overexpression vector, and cell viability was assessed at various time points. *E*, T-24 and 5637 cells were transfected with shWWP1 in combination with sh-NC or sh-HIPK3, and total cellular lysates were analyzed by Western blotting with the indicated antibodies. *F*, T-24 and 5637 wild-type cells were untreated as controls, while T-24 and 5637 WWP1-depleted cells were transfected with sh-NC or sh-HIPK3. Cell viability was measured at different time points. *G*, A xenograft tumor model was established in nude mice using T-24 cells with WWP1 and HIPK3 expression interference. Tumor size was recorded after 40 days of monitoring. *H*, tumor weight was measured. Data are presented as mean ± SD (n ≥ 3), ∗*p* < 0.05; ∗∗*p* < 0.01.

To investigate the clinical significance of the WWP1/HIPK3 axis in BC, the effects of WWP1 and HIPK3 on BCa cell viability were assessed. Knockdown of WWP1 significantly reduced BCa cell viability (Fig. 4*A*), while overexpression of wild-type WWP1, but not the inactive C890 A mutant, enhanced cell viability (Fig. 4*B*). Conversely, silencing HIPK3 promoted BCa cell viability (Fig. 4*C*), and overexpression of wild-type HIPK3, but not the D322 N kinase-inactive mutant, reduced cell viability (Fig. 4*D*). To further explore the functional relevance of HIPK3 in WWP1-deleted BCa cells, HIPK3 was silenced in WWP1 knockout BCa cells. The protein levels of HIPK3 in these cells were comparable to those in wild-type cells transfected with a negative control (Fig. 4*E*). Although WWP1 knockout cells exhibited reduced viability relative to their wild-type counterparts, HIPK3 silencing significantly reversed this effect (Fig. 4*F*). *In vivo*, T24 cells stably transfected with sh-NC, sh-WWP1, HIPK3 overexpression (OV), and sh-WWP1 + HIPK3 OV were subcutaneously injected into nude mice to establish xenograft models. Downregulation of WWP1 significantly inhibited tumor growth compared to the control group (Fig. 4, *G* and *H*). In contrast, HIPK3 overexpression promoted tumor growth (Fig. 4, *G* and *H*), and partially reversed the inhibitory effects of WWP1 knockdown on tumor growth (Fig. 4, *G* and *H*). These results suggest that the WWP1/HIPK3 axis regulates the proliferation of BCa cells both *in vitro* and *in vivo*.

### WWP1/HIPK3 axis mediates BCa cells’ chemosensitivity

**Figure 5:**
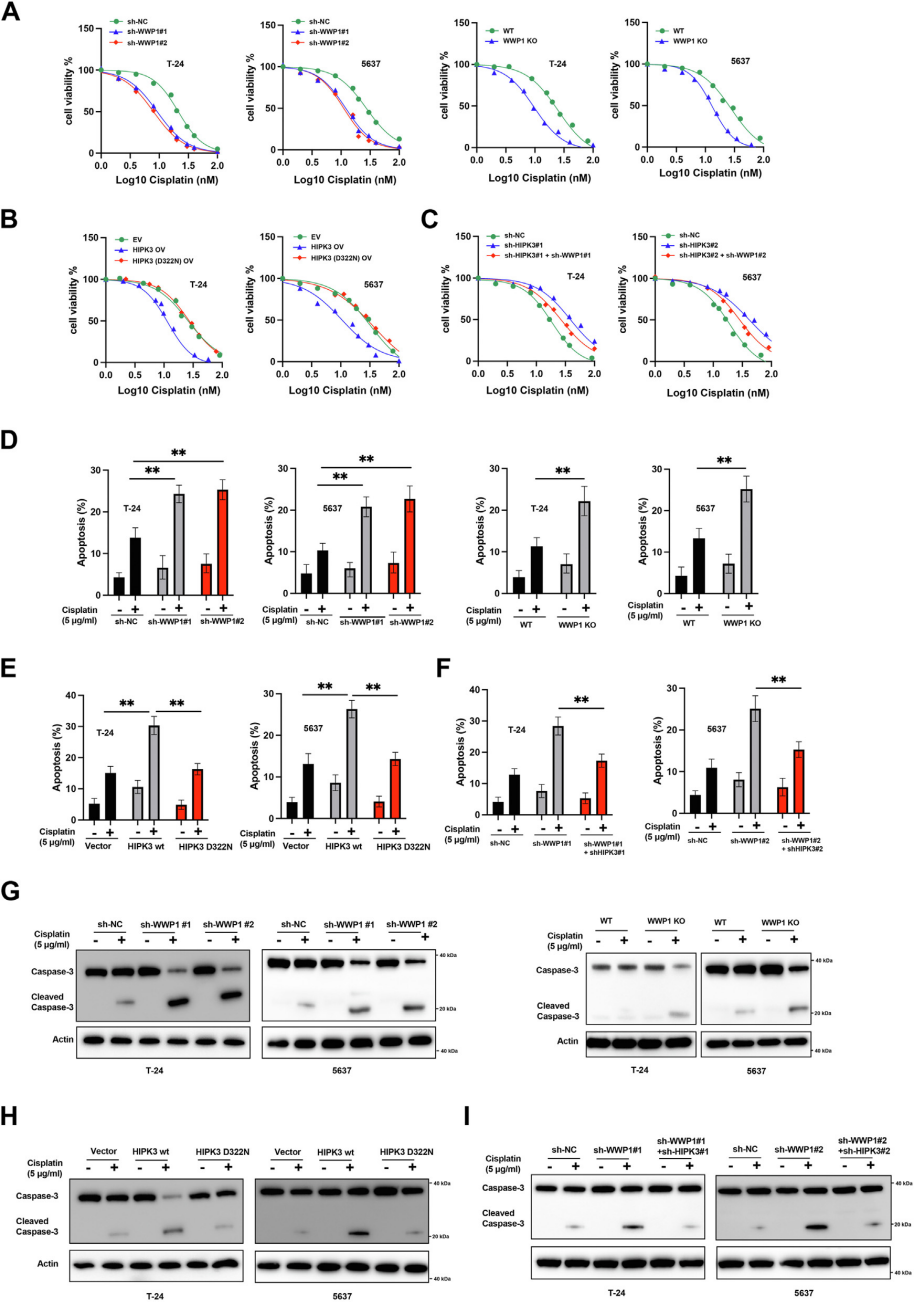
**WWP1/HIPK3 axis mediates BCa cell chemosensitivity to cisplatin**. *A*, T-24 and 5637 cells were transfected with sh-NC or shRNAs targeting WWP1, or sg-NC or sg-WWP1. Cells were treated with various doses of cisplatin, and cell viability was measured. *B*, T-24 and 5637 cells were transfected with an empty vector, HIPK3 overexpression vector, or HIPK3 mutant overexpression vector. Cells were treated with various doses of cisplatin, and cell viability was measured. *C*, T-24 and 5637 cells were transfected as indicated, treated with different doses of cisplatin, and cell viability was assessed. *D*, *E*, and *F*. T-24 and 5637 cells were transfected as indicated, treated with or without cisplatin, and apoptosis was measured by Annexin V/PI staining. *G*, *H*, and *I*. T-24 and 5637 cells were transfected as indicated, treated with or without cisplatin, and total cellular lysates were analyzed by Western blotting with the indicated antibodies. Data are presented as mean ± SD (n ≥ 3), ∗*p* < 0.05; ∗∗*p* < 0.01.

This study further examined whether the WWP1/HIPK3 axis influences BCa cell chemosensitivity. Inhibition of WWP1 significantly enhanced BCa cell sensitivity to cisplatin (Fig. 5*A*). Overexpression of wild-type HIPK3, but not the inactive mutant, increased BCa cell sensitivity to cisplatin (Fig. 5*B*). Silencing HIPK3 reduced chemosensitivity to cisplatin, and WWP1 inhibition reversed this effect (Fig. 5*C*). Annexin V staining revealed that WWP1 knockdown or depletion significantly enhanced cisplatin-induced apoptosis in BCa cells (Fig. 5*D*). Overexpression of wild-type HIPK3, but not the inactive mutant, also increased cisplatin-induced apoptosis (Fig. 5*E*). WWP1 knockdown promoted cisplatin-induced apoptosis, which was significantly reversed by simultaneous HIPK3 knockdown (Fig. 5*F*). Western blotting analysis of caspase-3 corroborated these findings from the annexin V staining assays (Fig. 5, *G*, *H*and *I*). These results collectively suggest that the WWP1/HIPK3 axis regulates the response of BCa cells to cisplatin.

### WWP1/HIPK3 axis affects JNK signaling pathway in BCa cells

**Figure 6:**
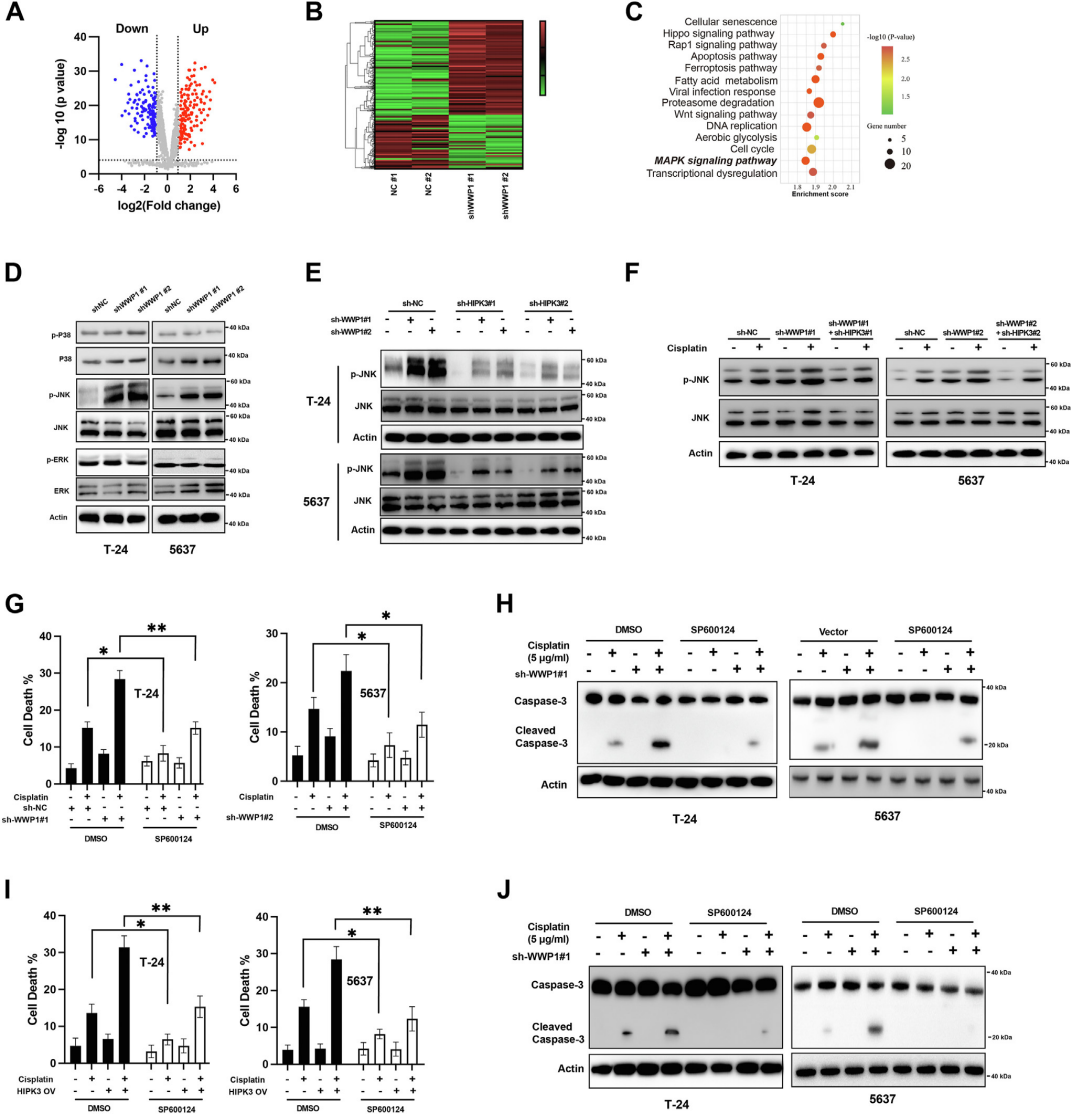
**WWP1/HIPK3 axis regulates JNK signaling in BCa cells**. *A*, a volcano plot reveals differential gene expression in T-24 cells following WWP1 downregulation. *B*, a heatmap displaying the differential expression of genes. *C*, Kyoto Encyclopedia of Genes and Genomes (KEGG) database enrichment analysis of affected pathways. *D*, T-24 and 5637 cells were transfected as indicated, and total cellular lysates were analyzed by Western blotting with the indicated antibodies. *E*, T-24 and 5637 cells were transfected as indicated, and total cellular lysates were analyzed by Western blotting with the indicated antibodies. *F*, T-24 and 5637 cells were transfected as indicated, treated with or without cisplatin, and total cellular lysates were analyzed by Western blotting with the indicated antibodies. *G*, T-24 and 5637 cells were pre-treated with or without SP600124, transfected with sh-NC or shRNAs targeting WWP1, and treated with or without cisplatin. Cellular death was measured. *H*, Cells were treated as described above, and activated caspase-3 levels were detected by Western blotting. *I*, T-24 and 5637 cells were pre-treated with or without SP600124, transfected with empty vector or HIPK3 overexpression vector, and treated with or without cisplatin. Cellular death was measured. *J*, cells were treated as described above, and activated caspase-3 levels were detected by Western blotting. Data are presented as mean ± SD (n ≥ 3), ∗*p* < 0.05; ∗∗*p* < 0.01.

To further elucidate the biological functions of the WWP1/HIPK3 axis, the effects of WWP1 knockdown on the transcriptome of BCa cells were assessed. T24 cells were transfected with either negative control shRNA (shNC) or sh-WWP1, followed by RNA-seq analysis. An average of 50 million paired-end 150-bp sequencing reads per sample were obtained. Differential expression analysis, using a log2 (fold change) > 1 threshold, identified 372 genes significantly up-regulated and 269 genes down-regulated following WWP1 knockdown compared to the negative control in T24 cells (Fig. 6*A*). Cluster analysis produced a heat map, grouping the samples into two primary clusters (Fig. 6*B*). KEGG enrichment analysis revealed that WWP1 knockdown affected several pathways, including the MAPK pathway (Fig. 6*C*), a frequently dysregulated pathway and a potential target in BCa (17). To validate this, the status of MAPKs was assessed by Western blotting. As shown in Figure 6*D*, WWP1 knockdown did not alter the levels of p-P38 and p-ERK but increased p-JNK levels in BCa cells. The potential role of HIPK3 in regulating JNK pathway activation upon WWP1 knockdown was next examined. Figure 6*E* shows that HIPK3 silencing markedly decreased p-JNK levels in BCa cells after WWP1 downregulation. Additionally, WWP1 knockdown significantly enhanced cisplatin-induced JNK signaling in BCa cells, an effect that was reversed by HIPK3 silencing (Fig. 6*F*). To explore whether the enhanced chemosensitivity following WWP1 knockdown was mediated through the JNK pathway, the specific JNK inhibitor SP600124 was applied. SP600124 significantly reduced the enhancement of cisplatin-induced cell death in BCa cells upon WWP1 inhibition (Fig. 6*G*). Western blotting confirmed that SP600124 mitigated the increased caspase-3 activation induced by cisplatin following WWP1 downregulation (Fig. 6*H*). Moreover, SP600124 attenuated cisplatin-induced cell death and caspase-3 activation caused by HIPK3 overexpression in BCa cells (Fig. 6, *I* and *J*). Collectively, these results suggest that the WWP1/HIPK3 axis regulates BCa cell chemosensitivity to cisplatin, at least in part, *via* the JNK signaling pathway.

### Myc regulates the expression of WWP1 in BCa cells

**Figure 7:**
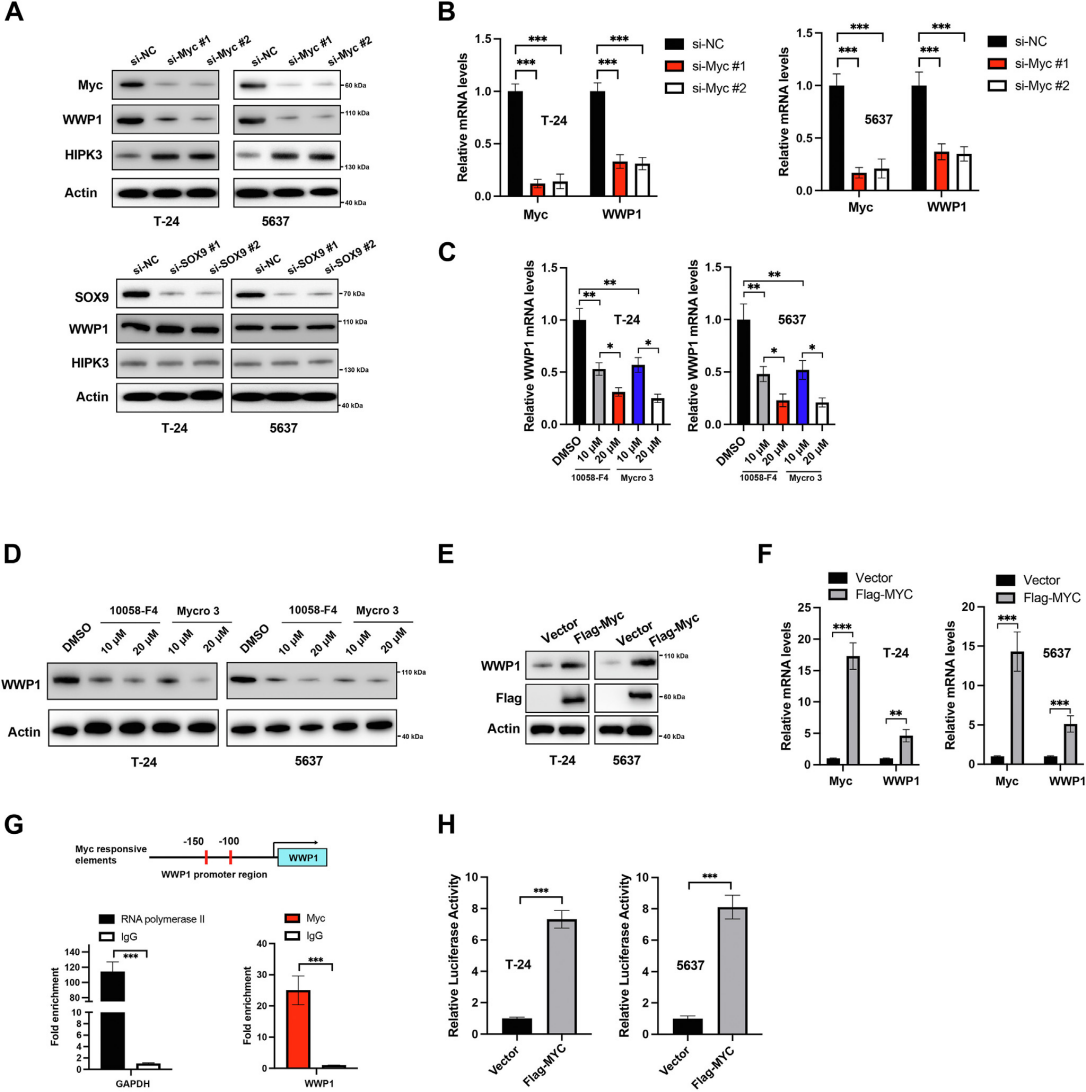
**Myc directly regulates WWP1 transcription in BCa cells.***A*, T-24 and 5637 cells were transfected with siRNAs targeting Myc or SOX9. Total cellular lysates were analyzed by Western blotting with the indicated antibodies. *B*, T-24 and 5637 cells were transfected with siRNAs targeting Myc or SOX9, and mRNA levels of Myc and WWP1 were measured by RT-PCR. *C*, T-24 and 5637 cells were treated with Myc inhibitors (10,058-F4, Mycro 3), and mRNA levels of WWP1 were measured by RT-PCR. *D*, T-24 and 5637 cells were treated with Myc inhibitors (10,058-F4, Mycro 3), and protein levels of WWP1 were measured by Western blotting. *E*, T-24 and 5637 cells were transfected with empty vector or Flag-Myc, and total cellular lysates were analyzed by Western blotting with the indicated antibodies. *F*, T-24 and 5637 cells were transfected with empty vector or Flag-Myc, and mRNA levels of Myc and WWP1 were measured by RT-PCR. *G*, the chromatin level of MYC at the WWP1 promoter region was assessed in T-24 cells. Fold enrichment of MYC relative to normal mouse IgG was quantified by ChIP assay, with RNA polymerase II enrichment at the GAPDH promoter region serving as a positive control. *H*, dual-luciferase activity assays were conducted to verify the binding of Myc to the WWP1 promoter region. Data are presented as mean ± SD (n ≥ 3), ∗∗*p* < 0.01; ∗∗∗*p* < 0.001.

To further elucidate the upstream mechanisms driving WWP1 upregulation in BCa cells, the role of Myc and SOX9, which have been shown to promote WWP1 expression at the transcriptional level, was explored (18, 19). Myc or SOX9 was silenced in BCa cells (Fig. 7*A*). Downregulation of Myc, but not SOX9, significantly reduced WWP1 protein levels while increasing HIPK3 protein levels in BCa cells (Fig. 7*A*). RT-PCR results confirmed that Myc silencing also decreased WWP1 mRNA levels in BCa cells (Fig. 7*B*). To further validate Myc's role in WWP1 upregulation, two specific Myc inhibitors (10,058-F4, Mycro 3) were applied. Treatment with either 10,058-F4 or Mycro 3 reduced both WWP1 mRNA and protein levels in BCa cells (Fig. 7, *C* and *D*). Overexpression of Myc, on the other hand, elevated WWP1 expression at both the protein and mRNA levels (Fig. 7, *E* and *F*). Chromatin immunoprecipitation (ChIP) and dual-luciferase reporter assays were then performed to determine whether Myc directly interacts with the WWP1 promoter. ChIP analysis revealed significant enrichment of Myc in the WWP1 promoter region, with RNA polymerase II binding to the GAPDH promoter region serving as a positive control (Fig. 7*G*). The dual-luciferase reporter assay showed that Myc overexpression significantly enhanced luciferase activity linked to the WWP1 promoter region (Fig. 7*H*). These results indicate that Myc acts as a transcription factor regulating WWP1 expression in T24 cells.

## Discussion

Growing evidence suggests that HIPK3 functions as a tumor suppressor in several cancers, including lung, gastric, hepatocellular, and renal cell carcinomas (7, 8, 9). However, the mechanisms underlying HIPK3 downregulation in various cancers remain poorly understood. Previous studies have indicated that HIPK3 mRNA levels are regulated by microRNAs such as miR-3174, miR-205-5p, and miR-146-5p (20, 21, 22). Despite this, the PTMs of HIPK3 remain largely unexplored. This study identifies E3 ligase WWP1 as a key mediator of HIPK3 ubiquitination and degradation. While the interaction between WWP1 and HIPK3 has not been previously reported, this study presents experimental evidence demonstrating that WWP1 binds to HIPK3 through its WW domain. Notably, Lysine 1187 was identified as the ubiquitination site in HIPK3, essential for its proteasomal degradation. Furthermore, the WWP1/HIPK3 axis regulates BCa cell chemosensitivity to cisplatin *via* modulation of the JNK signaling pathway. These mechanistic insights suggest that targeting the WWP1/JNK axis could serve as a promising therapeutic strategy for BCa.

HIPK3 has emerged as a tumor suppressor in various cancers. In prostate cancer, HIPK3-mediated FADD phosphorylation is crucial for FAS-induced apoptosis (23). Additionally, HIPK3 expression serves as a valuable biomarker for survival and prognosis in non-small cell lung cancer (9), and low HIPK3 expression correlates with poor prognosis in renal cell carcinoma (7). However, the mechanisms underlying HIPK3 downregulation in tumors remain largely undefined. Mass spectrometry combined with co-immunoprecipitation (Co-IP) revealed that HIPK3 interacts with the E3 ligase WWP1. Ubiquitination, a widespread PTM, involves the addition of ubiquitin to lysine residues of target proteins. This process is tightly regulated by E1, E2, and E3 enzymes. Dysregulation of E3 ligases has been shown to contribute significantly to tumorigenesis by inhibiting tumor suppressors and/or activating oncogenes (24). For instance, the E3 ligase ABLIM1 is upregulated in colorectal cancer and promotes tumor growth and metastasis through activation of the NF-κB signaling pathway (25). Another E3 ligase, RNF126, regulates PTEN ubiquitination and degradation, thereby promoting BCa progression both *in vitro* and *in vivo* (26). WWP1, a highly conserved protein, features an N-terminal C2 domain, four WW domains, and a C-terminal HECT domain (14). WWP1 is implicated in various human diseases, including neurological disorders, infectious diseases, diabetes, and cardiac atrophy (14, 27). The biological functions of WWP1 in various tumors remain contentious, as it can function both as an oncogene and a tumor suppressor. WWP1 promotes the ubiquitination and degradation of the oncogenic chemokine receptor CXCR4, thereby inhibiting breast cancer migration and metastasis (28). Additionally, upregulation of WWP1 has been shown to suppress malignant phenotypes in glioma cells *in vitro* and inhibit tumor growth *in vivo* (29). Identifying novel substrates is essential for elucidating WWP1's roles in tumor biology. The present study demonstrates that the WW domains of WWP1 mediate interactions with HIPK3. This finding aligns with previous reports highlighting the critical role of WW domains in WWP1's interactions with various proteins, including RNF11, ErbB4, and p27 (16, 30, 31). WWP1 targets a range of PY motif-containing substrates, such as Smad2, KLF5, and p63 (32). The PEST domain of HIPK3, which is proline-rich, facilitates binding between WWP1 and HIPK3, potentially explaining why HIPK3, despite lacking a PY motif, is still recognized by WWP1. This observation is consistent with prior studies indicating that certain WWP1 substrates, including p53, KLF2, and p27, lack a PY motif but possess a proline-rich domain (31, 33, 34). Notably, except for HIPK4, all HIPK family members share similar structural features. Therefore, WWP1's ability to induce degradation of HIPK3 may extend to HIPK1 and HIPK2, warranting further investigation in future studies.

A key discovery of this study is that the WWP1/HIPK3 axis regulates the JNK signaling pathway in BCa. JNK is pivotal in the development, metastasis, and invasion of various cancers, including BCa (35), and is linked to the response of cancer cells to chemotherapeutic agents. Cisplatin activates JNK in numerous cancer cells, and JNK inhibition results in chemoresistance (36). Consistent with prior reports, this study observed that cisplatin treatment activates JNK in BCa cells. Moreover, the WWP1/HIPK3 axis modulates JNK activation. Interestingly, HIPK3 appears to activate JNK independently of its kinase activity, as the inactive mutant form of HIPK3 was still capable of activation. One possible explanation is that HIPK3 may serve as a scaffold to recruit other kinases to activate JNK, as indicated by our LC-MS/MS results showing interactions between HIPK3 and various kinases. Notably, a previous study reported that JNK regulated HIPK3 expression in prostate carcinoma cells (23), suggesting the existence of a positive feedback loop between HIPK3 and JNK. However, in contrast to our findings, another investigation showed that HIPK3 inhibited JNK signaling in monocytes from a rat sepsis model induced by cecal ligation and puncture (37). This discrepancy could arise from differences in cell types or stimuli, warranting further exploration of the relationship between HIPK3 and JNK signaling in additional cell types.

In addition to the downstream effects of the WWP1/HIPK3 axis in BCa, the upstream factors that drive WWP1 upregulation in BCa were examined. Previous studies have identified transcription factors such as MYC, KLF2/5, and SOX9 as regulators of WWP1 expression (32). Among these, MYC is a well-established oncogene frequently dysregulated in various cancers, including BCa (38). Furthermore, aberrant activation of the Myc/WWP1 axis is often associated with cancer progression (18). Our findings provide new insights into Myc's role in regulating BCa cell proliferation and chemosensitivity.

Several limitations remain in this study. Firstly, the precise mechanism by which HIPK3 activates JNK signaling was not addressed, and this will be explored in future research. Secondly, the WWP1/HIPK3 axis may influence other signaling pathways that also contribute to BCa tumorigenesis, which warrants further clarification.

## Conclusion

In conclusion, this study demonstrated that WWP1 acts as an E3 ligase, facilitating the ubiquitination and degradation of HIPK3. The mechanisms underlying the WWP1-HIPK3 interaction were elucidated. The WWP1/HIPK3 axis modulates BCa cell proliferation and chemosensitivity through the regulation of JNK signaling. Additionally, MYC was identified as an upstream regulator of WWP1. These findings suggest that targeting the Myc/WWP1 axis may offer a potential therapeutic strategy for BCa treatment.

## Experimental procedures

### Cell lines and cell culture

Human urinary tract epithelial cell line (SV-HUC-1) and human BCa cell lines (T24, 5637, RT4, J82, BIU-87) were purchased from the American Type Culture Collection (ATCC, USA). Cell lines were authenticated by short tandem repeat profiling. All cells were maintained in RPMI1640 medium (Pricell) supplemented with 15% fetal bovine serum (Pricell) and 100 U of penicillin/streptomycin (Sigma). Cells were cultured in a humidified atmosphere with 5% CO_2_ at 37 °C. All cells were mycoplasma-free and routinely tested by morphological quality examinations and the growth profile.

### Chemicals and antibodies

Puromycin, cisplatin, 100,584-F4, Mycro 3, and SP600124 were ordered from Selleck Chemicals (USA).MG132 and CHX were ordered from Sigma. Primary antibodies, including anti-Flag, anti-HA, anti-WWP1, anti-actin, anti-ubiquitin, anti-phospho-P38, anti-phospho-JNK, anti-phospho-ERK, anti-P38, anti-JNK, and anti-ERK, were procured from Cellular Signaling Technology. Primary antibodies, including anti-caspase-3, anti-HIPK3, anti-SOX9, and anti-Myc, were procured from Abcam. The secondary antibodies were purchased from Abclonal (China).

### Plasmids, siRNA, shRNAs, CRISPR/Cas9, and transfection

pcDNA3.1-Flag-CMV, pcDNA3.1-HA-N vectors, and pRK5-Myc-Ubiquitin, pLVX-shRNA1 vectors were obtained from GenePharma BioTech. The coding regions of WWP1 and HIPK3 were subcloned into the vectors using the Homologous Recombination Kit (Norgen) according to the manufacturer’s instructions. PCR and cloning accuracy were confirmed by DNA sequencing. Mutants were generated using the QuikChange Site-Directed Mutagenesis Kit (Stratagene). The Myc-Ub K48 and K63 ubiquitination plasmids were generously provided by Prof. Yingfeng Liu from Fudan University. For WWP1 knockout *via* CRISPR/Cas9, sgRNA sequences were designed by RioBio Technology. siRNAs targeting Myc or SOX9 were ordered from GenePharma Ltd. CRISPR/Cas9 plasmids encoding WWP1 sgRNA and Cas9 were cloned into the lentiCRISPR v2-Puro vector (Addgene). Cells were transfected with plasmids containing or lacking sgWWP1 using Lipofectamine 2000 (Life Technologies) and selected with puromycin. Lentivirus for knockdown was produced by transfecting HEK293 T cells with the pHRSIN vector, packaging vectors pCMVR8.91 (gag/pol), and pMD.G using Lipofectamine 2000 (Life Technologies). Viral supernatant was collected 48 h post-transfection, filtered through a 0.22 μm filter, and stored at −80 °C. Cells were seeded at 5 × 10^4^ cells/well in 24-well plates and cultured overnight before adding 500 μl of viral supernatant to each well. Transfection efficiency was validated by Western blotting, and stable clones were selected by treating cells with puromycin (5 mg/ml). Sequences for siRNAs, shRNAs, and sgRNAs are provided in Table S1.

### Cell viability assay

Cell Counting Kit-8 (CCK-8) proliferation assay kit (Dojindo, Japan) was applied to measure the cellular viability. 96-well plates were seeded with 5 × 10^3^ cells per well. The cells underwent various treatments for different durations. 10 μl of CCK-8 solution was added to each well and incubated for 2 h. Absorbance was measured at 450 nm using a BioTek microplate reader, and the procedure was performed in triplicate.

### Measurement of apoptosis

Cellular apoptosis was assessed using the Annexin V-FITC staining kit (Beyotime) as previously described (39). In brief, cells were seeded into 6-well plate at a density of 1 × 10^6^ cells/well. After different treatments, cells were digested with trypsin and washed three times with ice-cold PBS. Annexin V-FITC and PI dyes were used to stain the cells following the protocol provided by the kit. Apoptotic cells were quantified using flow cytometry, and their proportions were analyzed with ImageJ software.

### Quantitative real-time PCR (qRT‒PCR)

RNA was purified from cells using TRIzol reagent (Life Technologies) after various treatments. DNase I (Beyotime Biotechnologies) was added and incubated for 20 min at 37°C to remove DNA. The reaction was halted by adding the Stop solution and incubating at 70°C for 10 min. High-Capacity cDNA Reverse Transcription Kit (Qiagen, USA) was used to synthesize the cDNA. Quantitative PCR was conducted with SYBR Supermix (Takara) on a QuantStudio Real-Time PCR system (Applied Biosystems). GAPDH mRNA levels were used as the internal control. Primers were listed in Table S2.

### RNA-sequencing

Total RNA was isolated from the cells as described previously. RNA sequencing was performed on an Illumina HiSeq X-10 platform by Shanghai HiBio Technology. Sequencing libraries were prepared using the TruSeq Library Prep Kit (Illumina) following the manufacturer’s instructions and sequenced on the HiSeq7500 platform (Illumina). Reads were aligned to the human hg19 genome, and gene expression values were calculated using edgeR by counting the mapped reads. *p*-values were adjusted using the Benjamini-Hochberg method to control the false discovery rate. Genes with significant differential expression (|log2(Fold Change)| > 2, *p* < 0.05) were analyzed through KEGG (Kyoto Encyclopedia of Genes and Genomes) pathway analyses.

### Immunoprecipitation-mass spectrometry (IP-MS)

To identify potential proteins interacting with HIPK3 in BCa cells, IP-MS was conducted. Flag-HIPK3 vector was transfected into 293K cells for 24 h using Lipofectamine 2000 (Life Technologies). Cells were lysed using NP40 lysis buffer containing protease inhibitor (Beyotime Technologies). The lysates were centrifuged to obtain the supernatant, to which 25 μl of anti-FLAG antibody-conjugated agarose beads (Sigma) were added. The precipitates were eluted with 150 ng/ml FLAG peptides in TBS. Samples were analyzed using liquid chromatography–tandem mass spectrometry (LC-MS/MS) by BiotechPack Ltd.

### Measurement of protein half-life

Culture medium was supplemented with CHX to a final concentration of 50 μg/ml prior to various treatments. Cells were then subjected to these treatments and collected at specified time points. The cells were lysed on ice for 30 min, followed by protein analysis using 10% SDS-PAGE and transfer to PVDF membranes. Target proteins were quantified using ImageJ software (version 1.61, NIH), and protein levels were normalized relative to the loading control.

### Co-immunoprecipitation (co-IP)

For Co-IP analysis, cells were lysed with co-IP lysis buffer (Beyotime Technologies) containing protease inhibitors and PhosSTOP (Roche). Protein immunoprecipitation was performed by incubating 1 μl of specific antibodies bound to Sepharose G beads (Abcam), with IgG used as a negative control. The protein was eluted under reducing and denaturing conditions using 5 × SDS sample buffer at 95°C for 5 min, followed by immunoblotting analysis.

### Western blotting

Cells were collected post-treatment and lysed in RIPA buffer (Beyotime Biotechnologies) on ice for 30 min. Total protein was separated using a 10% SDS-PAGE gel (Epizyme Biotechnologies) and transferred to a PVDF membrane (Merck). The membrane was blocked at room temperature for 15 min using a Western Rapid Kit (GeneScript). The membrane was incubated with primary antibodies diluted in the blocking buffer overnight at 4°C.The membranes were washed thrice with TBS-T and incubated with secondary antibodies at room temperature for 1 h, followed by visualization using an enhanced chemiluminescence (ECL) kit (Beyotime Technologies).

### Chromatin immunoprecipitation (ChIP)

ChIP was conducted using the ChIP Kit-One Step kit (Abcam) according to the manufacturer’s protocol. Cells were fixed with formaldehyde for 10 min, and chromatin was extracted using the Chromatin Extraction Kit (Abcam) as per the manufacturer's instructions. The extracts were incubated overnight at 4°C with anti-Myc or IgG antibodies bound to protein beads. Immunoprecipitated DNA was quantified by real-time PCR. Genomic DNA enrichment was calculated as the percent recovery relative to the input using the formula: Percent Input = 2% × 2^∧^(C[T] 2% Input Sample – C[T] IP Sample).

### Dual-luciferase activity assay

The WWP1 promoter region was amplified by PCR and subcloned into the pmirGLO vector (Hangzhou KeLei Biotechnology Ltd, China). BCa cells were seeded into 24-well plates at a density of 10^4^ cells per well. After 24 h, cells were co-transfected with pmirGLO-WWP1 and either an empty vector or a Flag-Myc overexpression vector. Luciferase activity was measured using the dual-luciferase reporter assay kit (Promega), and relative luciferase activity was determined by the ratio of firefly to Renilla luciferase activity. The experiment was performed in triplicate.

### Ni-NTA pull-down assay

After different transfections, cells were treated with MG132 (10 μM) overnight and lysed with Buffer A (6 M guanidine-HCl, 0.1 M Na2HPO4/NaH2PO4, pH 8.0, and 10 mM imidazole). The cellular lysates were incubated with Ni-NTA Sepharose beads (Sigma-Aldrich) for 3 h at 4°C. The beads were washed once with Buffer A, twice with Buffer A/TI (1 volume of Buffer A and 3 volumes of Buffer TI [20 mM imidazole and 25 mM Tris-HCl, pH 6.8]), and three times with Buffer TI. Subsequently, the pull-down proteins were subjected to SDS-PAGE analysis.

### *In vitro* ubiquitination assay

HEK293 T cells were first transfected with HA-WWP1, which was purified *via* HA affinity precipitation. Then, 1 μg of bacterially purified His-tagged HIPK3 protein was incubated with the purified WWP1 at 37°C for 60 min in 50 μl of reaction buffer (50 mM Tris, pH 7.5, 5 mM MgCl2, and 2 mM DTT), containing purified UBE1 (100 nM, Boston Biochem), UbcH13/Uev1a (1 μM, Boston Biochem), His-ubiquitin, His-K48O-ubiquitin, or His-K63O-ubiquitin (UBbiotech). The reaction mixtures were heated, then diluted with buffer (0.05% Triton X-100, 0.1 M Na2HPO4/NaH2PO4, 10 mM imidazole, pH 8.0) for the purification of ubiquitinated HIPK3 using Ni-NTA beads (Qiagen). Eluted proteins were analyzed by Western blotting.

### Mouse xenograft model

BALB/c nude mice (4 weeks old) were purchased from Hangzhou ZhiAo Biotechnology and housed in a pathogen-free environment. The corresponding BCa cell model was subcutaneously injected into the dorsal region of the nude mice to establish the xenograft tumor model. The following groups were set up: sh-NC (n = 5), shWWP1 (n = 5), OV-HIPK3 (n = 5), and sh-WWP1 + OV-HIPK3 (n = 5). Tumor size was measured every 5 days using calipers, and tumor volume was calculated using the formula: L × W^2^ × 0.5, where L is the longest diameter and W is the shortest diameter. At the study's conclusion, mice were euthanized by cervical dislocation under anesthesia, and the tumors were removed and measured. All animal experiments were approved by the Committee on the Ethics of Animal Experiments of Ningbo University.

### Statistical analysis

Prism 9.0 software (TreeStar) was used for statistical analyses. The findings are presented as the mean ± SD. For between-group comparisons, a two-tailed Student’s *t* test for two samples with unequal variance was used. Multiple group comparisons were conducted using one-way ANOVA followed by Tukey’s *post hoc* test. A two-tailed *p* < 0.05 indicated statistical significance.

## Data availability

Data are available from the corresponding author upon reasonable request.

## Supporting information

This article contains supporting information.

## Conflict of interest

The authors declare that they have no conflicts of interest with the contents of this article.
