# Supplementary material for: WWP1 mediates the ubiquitination and degradation of HIPK3 in bladder cancer cells
Source: J Biol Chem. 2025 Apr 23;301(6):108528. doi: 10.1016/j.jbc.2025.108528 (PMC12155759; doi:10.1016/j.jbc.2025.108528)
Supplement: Supplementary Table 1 and 2 [file mmc2.docx]

**Supplementary Table S1.** siRNAs, shRNAs and sgRNA oligonucleotides

| **Target genes** | **Sequences (5′-3′)** |
| --- | --- |
| WWP1(shRNA#1) | Forward: AAGGTGGATCCTGAACGTGAC |
|  | Reverse: GTCACGTTCAGGATCCACCTT |
| WWP1  (shRNA#2) | Forward: AAGCAGATGTCCAGTGCAAGC |
|  | Reverse: GCTTGCACTGGACATCTGCTT |
| WWP1 (sgRNA) | Forward: CACCGGCTGAAGACCTGCAGATCTGT |
|  | Reverse: AAACACAGATCTGCAGGTCTTCAGCG |
| Negative control  (shRNA) | Forward: CCCCTCCGGGAAACTGTGGCGTGATGGCC |
|  | Reverse: TATCGAAAATTGTCTAAGACTTTGGA |
| HIPK3 (shRNA#1) | Forward: AAGCTACGTGATCCAGAACTAC |
|  | Reverse: GTAGTTCTGGATCACGTAGCTT |
| HIPK3 (shRNA#2) | Forward: AATACAGCTGGTACAAGCTCAT |
|  | Reverse: ATGAGCTTGTACCAGCTGTATT |
| Myc (siRNA#1) | Forward: AATGGTCATTTGATTTTTATT |
|  | Reverse: TAAAAATCAAATGACCATTTT |
| Myc (siRNA#2) | Forward: AAGACGACGAGAACAGTTATT |
|  | Reverse: TAACUTGTTCTCGTCGTCTTTT |
| SOX9 (siRNA#1) | Forward: AATGGCCTACGATGAGCTACTT |
|  | Reverse: GTAGCTCATCGTAGGCCATTTT |
| SOX9 (siRNA#2) | Forward: AAGACCATCGAGCTGAAGACTT |
|  | Reverse: GTCTTCAGCTGATGGTCTTTT |
| Negative control  (siRNA) | Forward: TTCTCCGAACGTGTCACGTTT |
|  | Reverse: ACGTGACACGTTCGGAGAATT |

**Supplementary Table S2.** Sequences of real-time PCR primers

| **Gene name** | **Primer** | **Sequences (5′-3′)** | **Annealing temperature** |
| --- | --- | --- | --- |
| Myc | Forward | CCTGGTGCTCCATGAGGAGAC | 60°C |
|  | Reverse | CAGACTCTGACCTTTTGCCAGG |  |
| WWP1 | Forward | CAGACGGAGCAGAGCAAGAAC | 60°C |
|  | Reverse | GCTGTCCTTGTTGCTGTAGGT |  |
| GAPDH | Forward | GAAGGTGAAGGTCGGAGTCA | 59°C |
|  | Reverse | CATGGGTGGAATCATATTGGAA |  |
